# Individual and environmental determinants of body mass index trajectories: results from a longitudinal study in Southern Sweden

**DOI:** 10.1186/s12889-026-27378-1

**Published:** 2026-04-17

**Authors:** Pauline Rebouillat, Kristoffer Mattisson, Giedre Gefenaite, Per-Olof Östergren, Peter M. Nilsson, Jonas Björk

**Affiliations:** 1 Division of Occupational and Environmental Medicine, Lund University, Lund, Sweden; 2 Department of Health Sciences, Faculty of Medicine, Lund University, Lund, Sweden; 3 Social Medicine and Global Health, Lund University, Malmö, Sweden; 4 Department of Clinical Sciences, Lund University, Malmö, Sweden; 5 Department of Internal Medicine, Skåne University Hospital, Malmö, Sweden; 6 Clinical Studies Sweden, Forum South, Skåne University Hospital, Lund, Sweden

**Keywords:** greenspaces, neighbourhood, obesity, sociodemographic factors, environmental epidemiology

## Abstract

Exposure to natural environments is thought to benefit mental and physical health, but current evidence is mixed, and underlying mechanisms remain unclear. Considering the joint biodiversity, climate, and health crises, a better understanding of the interplay between individuals and their environment is imperative. This study aimed to (1) identify Body Mass Index (BMI) trajectories in the Scania Public Health Cohort (SPHC), (2) characterise trajectories in terms of sociodemographics, lifestyle, health and living environments, (3) study the associations between natural dimensions of residential environment and BMI trajectories. The SPHC was established in southern Sweden in 2000. Participants (*n* = 13 581 at baseline, 18–80 years old) responded to four surveys (2000–2016), including sociodemographic, lifestyle and health questions. Residential coordinates were linked to the Scania outdoor Environment Database, which comprised perceived sensory dimensions of residential areas, eight dimensions of the outdoor environment that have previously been identified as important to support people’s health. An adapted version of the Perceived Sensory Dimension Score (PSD3-score) was computed by summing up three dimensions related to the natural environment. Sex-specific BMI trajectories, identified using group-based trajectory modelling, were compared using adjusted multinomial regression. Five BMI trajectories were identified with similar shapes in men and women. Two trajectories started in the normal/overweight categories and were relatively stable over time, while the other three were close to or above the commonly used threshold for obesity. Overall, more favourable socioeconomic and lifestyle factors were observed within non-obese trajectories. The PSD3-score varied geographically across Scania, but for the selected environmental characteristics, little differences were observed in relation to BMI trajectories. For this population, our findings suggest that lifestyle and socioeconomic factors are more important than outdoor environment features for the long-term bodyweight and obesity development.

**Supplementary Information:**

The online version contains supplementary material available at 10.1186/s12889-026-27378-1.

## Introduction

Obesity is a global public health burden, which concerned 13% of the world adult population in 2016 [1]. An increasing trend is observed in many parts of the world [2]. In 2019, the Lancet Commission considered obesity as part of “The Global Syndemic”, together with malnutrition and climate change, constituting a major challenge for human health, environment and the planet in the 21st century [3]. Obesity is defined as abnormal or excessive fat accumulation, corresponding to a Body Mass Index (BMI) over 30 kg/m^2^. It is a major risk factor for many non-communicable diseases (cardiovascular diseases, diabetes, cancers, etc.), but also for some communicable diseases, as recently exposed during the COVID-19 pandemic [4–6]. Two main measures to slow down the increasing overweight and obesity trend are physical activity and diet. While these determinants of obesity are well documented, non-traditional risk factors such as environmental factors have been less investigated [7]. In the context of the joint biodiversity, climate, and health crises, studying interactions between people’s environment and their health becomes even more important. Indeed, exposure to natural environments is thought to have beneficial effects on mental stress through restoration [8] and on physical health through the physical activity pathway [9]. Greenspace and greenness have been studied in relation to different types of physical health outcomes, including obesity and cardiovascular diseases [10]. However, metrics and definitions vary considerably between studies in the literature [10], making it difficult to compare studies and to determine optimal measures. In this context, area-aggregated perceived sensory dimensions (PSDs) – eight dimensions of the outdoor environment which have been identified as important to support people’s health [11] – are thought to represent a compromise between objective and subjective measures and might be more likely to capture important effects on health-related behaviours [12].

Different approaches have been used previously to study obesity and BMI in relation to various risk factors. One promising approach is to use Group-Based Trajectory Modelling (GBTM) in order to model long-term variations [13, 14]. This approach has the advantage of capturing BMI variation over time, assuming that there is heterogeneity in the population, unlike more classical methods like mixed-effect modelling or averaged measures. This approach offers the opportunity to identify and target specific subgroups, which are of interest from a public health perspective.

In the present longitudinal cohort study, we aimed at (1) identifying different BMI trajectories in the Scania Public Health Cohort (SPHC); (2) characterising the identified trajectories in terms of sociodemographic and lifestyle factors, health and living environment; and (3) studying associations between natural dimensions of residential environment and BMI trajectories. We hypothesized that participants living in residential environments with natural elements perceived as serene, wild and diverse (combined into a PSD3-score) would have healthier BMI trajectories. We also expected more favourable socioeconomic and lifestyle factors in healthier BMI trajectories, as well as positive correlations between favourable socioeconomic and lifestyle factors and a higher PSD3-score.

## Methods

### Population

The SPHC was initiated in 1999–2000, in the region of Scania, southern Sweden [15]. Individuals living in Scania were invited to answer a questionnaire sent by mail, comprising 120 questions about sociodemographic characteristics, living and working conditions, health behaviours and self-rated health. Similar questionnaires were sent out again in 2005, 2010, and 2016 to all those who had participated in the baseline survey and were still alive and residing in Scania. Younger individuals, ages 18–34, randomly selected from the general population, were invited in 2010 and 2016. The SPHC recruited 15 916 participants over the four waves.

### Sample selection

A flowchart for the sample selection is presented in Supplementary Figure S1. For the present study, participants aged 72 years or older at baseline were excluded, and participants were censored during follow-up when they turned 76. Participants aged 71 or younger having at least 2 uncensored BMI measures were included (*N* = 10 046).

### Outcome

Weight (kilograms) and height (meters) were self-reported by participants at each time point. BMI (kg/m^2^) was calculated at each time point as: weight (kilograms)/(height (meters))^2^. BMI categories are named following the WHO definitions: <18.5 kg/m² as underweight, between 18.5 and 24.9 kg/m² as normal weight, between 25.0 kg/m² and 29.9 kg/m² as overweight, and ≥ 30.0 kg/m² as obesity [1].

### Exposure assessment

SPHC participants were matched to the Scania outdoor environment (ScOut) database using geographical coordinates corresponding to their residential location at the time of their first available BMI measurement and corresponding to 1 × 1 km squares. The ScOut database is an environmental exposure database for the Scania Region (Southern Sweden) characterizing the outdoor living environment within 5–10 minutes’ walk from the participants’ residences [16]. It consists of aggregated assessments of PSDs, previously identified as qualities of the outdoor environment potentially beneficial for people’s health [11], collected during 2008–2019 from two other public health surveys and two population-based cohort studies (the present SPHC and BIG3). Other items such as noise and air pollution are also available, as described in details elsewhere [16]. The assessments for each item were aggregated by 1 × 1 km area units. The proportions of positive assessments for each item were then estimated in each 1 × 1 km area unit using a multilevel ecometric model with individual and area levels, adjusted for demographic, socioeconomic characteristics, and seasonality. For this study, three PSDs related to natural environments were selected: serene, natural, and diverse, corresponding respectively to the following questions: “*Nature in the area where I live is quiet*,* one can hear nature’s own sound*”, “*Nature in the area where I live has nature that is wild and fascinating*”, and “*Nature in the area where I live has a large diversity of animal and plant species*”. Based on previous studies [17, 18], these three items were standardized and summed up to obtain a PSD sum score, hereafter named “PSD3-score”.

### Covariates

Covariates corresponded to the participants’ first available BMI measure.

Economic strain was used as a marker of socioeconomic position. Participants answered to the question “*How often in the last 12 months have you had difficulty managing your bills (rent*,* electricity*,* telephone*,* interest*,* repayments*,* insurance*,* etc.)?*”. Economic strain was considered absent when the answer was “never” or “occasionally” and present when the answer was “about half of the months” or “every month”. Country of birth was classified in two categories: participants born in Denmark, Finland, Iceland, Norway and Sweden, considered as born in a Nordic country and participants born in another country. Education level was categorized in three categories: primary education (less than 10 years), secondary education (10–12 years) and university or college (more than 12 years). Housing type was dichotomized into: “villa or chain-house” and “other”. Relation to employment was classified into 5 categories: home worker, employee, pensioner, student, and unemployed. Housing ownership was divided into three categories: “ownership, condominium or lease”, “condominium with paid contribution to the housing legal association” and “tenancy”.

General health was self-rated by participants on a 5-point scale ranging from “Very bad” to “Very good. Leisure physical activity comprised 4 levels: sedentary, moderate exercise, regular exercise, hard-training or competitive sports. Long-term illness corresponded to the declared presence of a long-term illness or disability (yes/no). Smoking habits were defined as never smoker, past smoker and current smoker based on the combination of two questions in the questionnaire.

Urban or rural settings of the participants’ residences were assessed based on Statistics Sweden’s definition of an urban area [19]. Neighbourhood coherence, neighbourhood safety and recreational possibilities, available through ScOut database were used as additional outdoor environment characteristics.

To account for cohort effect and potential age-dependent exposure effect, results are presented stratified in three age groups: 18–39 years old, 40–59 years old and more than 60 years old.

### Statistical analysis

Data management and statistical analyses were performed using SAS (version 9.4; SAS Institute, Inc.) and R (version 4.2.1).

GBTM was performed using SAS Proc TRAJ [13] and R lcmm package 2.0.0 version [20]. Models were compared using LCTMtools package [21]. Participants’ characteristics were mapped using ArcGIS (version 10.5.1). The GBTM was conducted separately for men and women, using age as timescale. Following Lennon *et al.’s* framework [14], the shapes of the residuals were firstly examined in a model with no random effects. A quadratic random effects model was chosen. The optimal number of trajectories was determined by comparing Bayesian Information Criterion (BIC) for models from 2 to 7 groups. As BIC decreased continuously with increasing number of groups, other adequacy measures were also used. Detailed criteria for the model selection are presented in Supplementary Table S1.

Logistic regression analysis stratified by sex and age group at baseline was used to study the associations between the natural outdoor living environment at baseline and BMI trajectories. Further adjustments were made for baseline covariates: individual age, economic strain, education level, birth in a Nordic country, housing type, relation to employment and residence (urban or rural). Neighbourhood coherence, neighbourhood safety and recreational possibilities were used as additional adjustments in a final model. Odds ratios and 95% confidence intervals were reported for a one-unit increase of the PSD3-score.

## Results

### Sociodemographic characteristics

Baseline sociodemographic characteristics of the participants are presented in Supplementary Table S2. The sample comprised 10 046 participants (45% of men and 55% of women). Participants were mostly included in 2000 (95% of participants). At baseline, on average women were 44.5 years old (SD 14.3) and men were 45.5 years old (SD 14.3). Average BMI at baseline was 24.2 kg/m^2^ (SD 3.9) in women and 25.6 kg/m^2^ (SD 3.4) in men. Most participants did not have difficulties managing their bills (89%), were born in a Nordic country (92%) and lived in urban areas (81%). University or college was the most represented educational level (40%). The most declared physical activity category was “moderate exercise” (60%), and a large proportion of participants had never smoked before (49%).

### Description of BMI trajectories

**Fig. 1:**
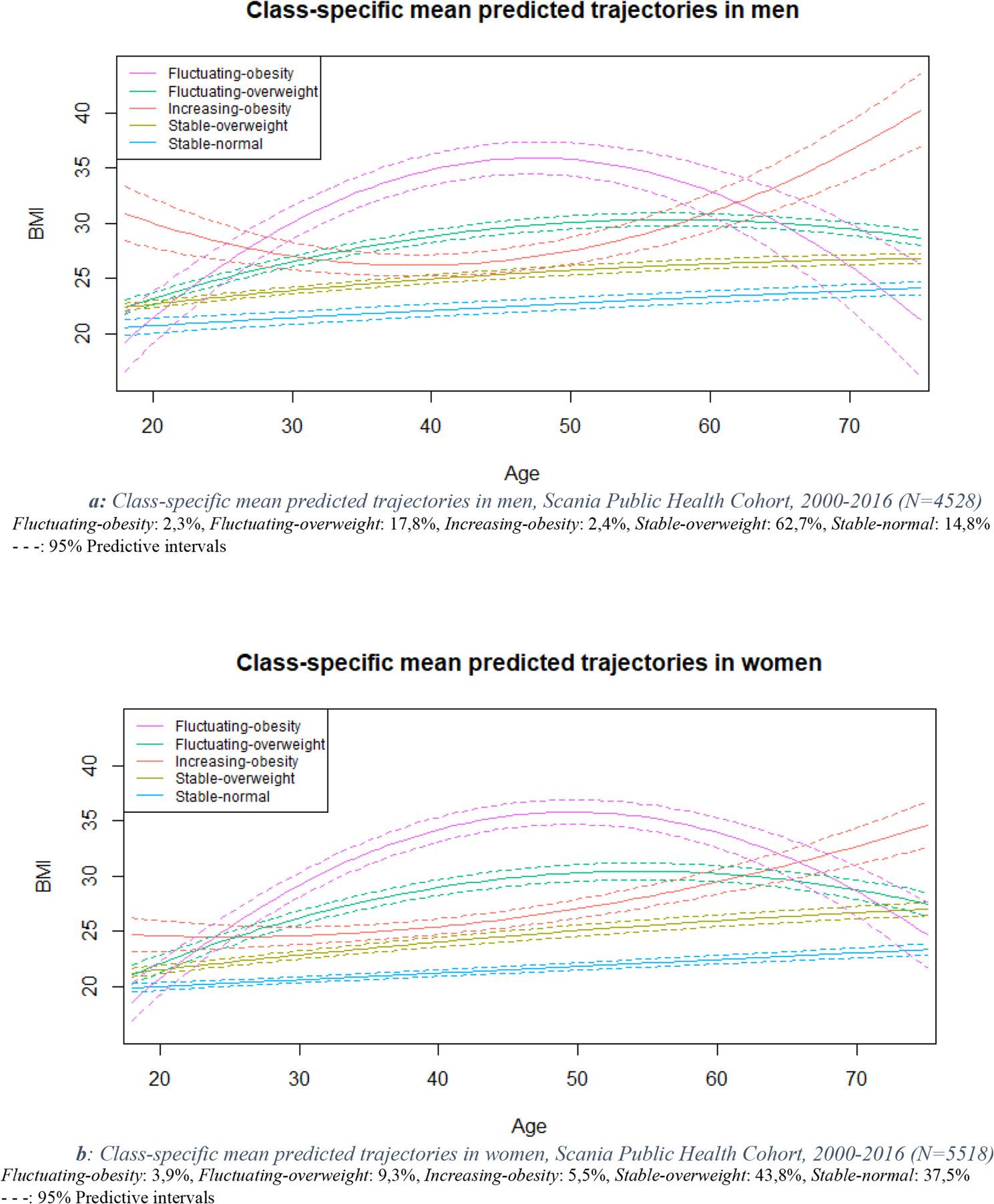
Class-specific mean predicted trajectories in men and women, Scania Public Health Cohort, 2000-2016

Five BMI trajectories were identified among men and among women, with similar shapes, as shown in Fig. 1. Baseline characteristics across trajectories and stratified by age are presented in Supplementary Tables S3 and S4. The *stable-overweight* trajectory comprised the most individuals both for men (63%) and women (44%). This linear-shaped trajectory started in between the normal and overweight categories and shifted towards the overweight category. Men and women (respectively 15% and 38%) belonging to the *stable-normal* trajectory started in the normal BMI category and stayed in this category while experiencing a slow increase. In the *increasing-obesity* trajectory (representing 6% of women and 2% of men) participants started in the overweight category, experienced a stable or decreasing period until midlife followed by an increase towards the obesity category. Men (2%) and women (4%) belonging to the *fluctuating-obesity* trajectory started in the normal weight category, experienced an increase until midlife and then experienced a decrease towards the normal weight category. The *fluctuating-overweight* trajectory was of similar shape with a more modest increase and decrease. To facilitate description and interpretation, the *stable-normal* and *stable-overweight* trajectories were merged together as *non-obese* trajectories and the three other as *obese* trajectories.

Baseline characteristics stratified by age using the merged trajectories are presented in Table 1. Different socioeconomic and lifestyle factors were observed across the trajectories. The highest proportions of university education and lowest proportions of sedentary lifestyle were found in the *non-obese* trajectories. Economic strain was higher in the *obese* trajectories and the highest in the *increasing-obesity* trajectory. Proportions of men and women declaring a long-term illness or disability were higher in the *obese* trajectories and the highest in the *fluctuating-obesity* trajectory. More current smokers were found in the *obese* trajectories, with highest proportions in the *increasing-obesity* trajectory, in both men and women.

**Table 1:** Baseline sociodemographic characteristics of participants across grouped trajectories stratified by sex and age, Scania Public Health Cohort, Sweden, 2000–2016 (*N* = 10046)

	Men						Women					
	18–39 years (*N* = 1628)		40–59 years (*N* = 2007)		60 + years (*N* = 893)		18–39 years (*N* = 2140)		40–59 years (*N* = 2410)		60 + years (*N* = 968)	
Trajectories	Non- obese	Obese	Non- obese	Obese	Non- obese	Obese	Non- obese	Obese	Non- obese	Obese	Non- obese	Obese
N	1107	521	1620	387	782	111	1619	521	1993	417	876	92
Age (years) T1, Mean (SD)	29.3	29.5	50.3	49.5	64.6	63.7	29.3	29.4	49.8	49.2	64.8	64.6
	6.2	6.0	5.5	2.3	3.2	2.8	6.1	6.0	5.6	5.9	3.2	3.0
BMI (kg/m^2^) T1, Mean (SD)	23.3	27.7	24.9	30.1	25.5	31.6	21.9	27.3	23.4	30.1	24.9	31.7
	2.1	3.9	2.3	3.3	2.5	3.7	2.2	3.9	2.6	4.4	3.0	4.1
Economic strain, n	1086	514	1600	380	762	107	1587	508	1962	408	837	89
Economic strain, %												
Yes	9.85	14.79	7.06	13.42	3.54	7.48	12.98	18.11	7.70	20.34	3.46	6.74
Born in a Nordic country, n	1102	519	1607	383	779	110	1606	518	1972	415	864	91
Born in a Nordic country, %												
No	8.17	10.02	8.03	9.92	6.16	6.36	9.03	6.76	6.85	12.29	5.32	3.30
Education level, n	1092	511	1593	374	748	105	1587	510	1930	407	823	80
Education level, %												
Primary	4.30	9.59	30.57	37.97	55.75	66.67	4.79	6.27	25.70	33.17	61.12	63.75
Secondary	48.81	56.16	25.24	29.68	12.70	13.33	44.05	53.92	27.46	32.68	15.43	15.00
University/college	46.89	34.25	44.19	32.35	31.55	20.00	51.17	39.80	46.84	34.15	23.45	21.25
Relation to employment, n	1050	493	1536	369	706	102	1519	482	1872	380	794	80
Relation to employment, %												
Home worker	1.24	1.42	1.04	3.52	1.98	1.96	6.25	7.05	3.69	6.05	4.91	3.75
Employed	68.29	71.81	90.95	81.84	36.54	34.31	58.20	57.68	83.92	70.00	26.45	35.00
Pensioner	0.67	1.01	3.19	6.50	57.22	53.92	0.79	1.66	5.61	11.05	63.60	58.75
Student	24.67	17.65	1.11	2.98	0.00	0.00	29.16	23.65	2.88	5.26	0.00	0.00
Unemployed	5.14	8.11	3.71	5.15	4.25	9.80	5.60	9.96	3.90	7.63	5.04	2.50
Physical activity, n	1091	513	1581	375	753	107	1592	513	1926	409	842	86
Physical activity, %												
Sedentary	13.66	23.98	12.84	23.20	9.03	20.56	13.07	16.76	11.84	24.69	8.67	25.58
Moderate exercise	43.63	47.17	64.90	63.47	76.36	74.77	52.70	60.82	64.12	65.28	77.08	68.60
Regular exercise	30.25	19.10	20.62	12.53	14.34	4.67	30.84	20.47	23.68	9.54	14.01	5.81
Hard-training/competitive	12.47	9.75	1.64	0.80	0.27	0.00	3.39	1.95	0.36	0.49	0.24	0.00
General health, n	1105	520	1613	385	781	110	1617	519	1979	417	873	92
General health, %												
Very good	30.05	22.50	22.44	17.40	18.31	7.27	26.41	16.96	18.54	11.03	16.38	13.04
Good	54.66	54.23	52.57	50.39	55.31	41.82	54.17	52.02	51.54	45.08	50.86	46.74
Fairly good	13.12	18.85	20.21	24.68	22.54	40.00	15.28	24.86	23.75	31.41	28.18	33.70
Bad	1.72	3.65	4.03	6.75	3.59	10.91	3.53	5.39	5.41	10.31	3.89	6.52
Very bad	0.45	0.77	0.74	0.78	0.26	0.00	0.62	0.77	0.76	2.16	0.69	0
Long-term illness or disability, n	1067	504	1568	368	685	91	1566	509	1899	386	722	79
Long-term illness or disability, %												
Yes	20.62	26.19	26.08	33.42	31.97	46.15	21.52	27.50	27.75	40.93	38.23	45.57
Smoking habits, n	1086	507	1591	380	758	108	1581	513	1951	410	844	87
Smoking habits, %												
Never smoker	68.60	62.33	39.79	30.26	39.84	33.33	61.54	55.36	41.93	38.05	56.52	48.28
Past smoker	16.21	21.50	38.78	42.63	43.67	55.56	18.98	19.88	31.37	34.39	29.38	35.63
Current smoker	15.19	16.17	21.43	27.11	16.49	11.11	19.48	24.76	26.70	27.56	14.10	16.09

*BMI* Body Mass Index, *SD* Standard Deviation

Non-obese trajectories correspond to *Stable-normal* and *stable-overweight* trajectories

Obese trajectories correspond to *Fluctuating-overweight*, *increasing-obesity* and *fluctuating-obesity* trajectories

### Spatial variation

**Fig. 2:**
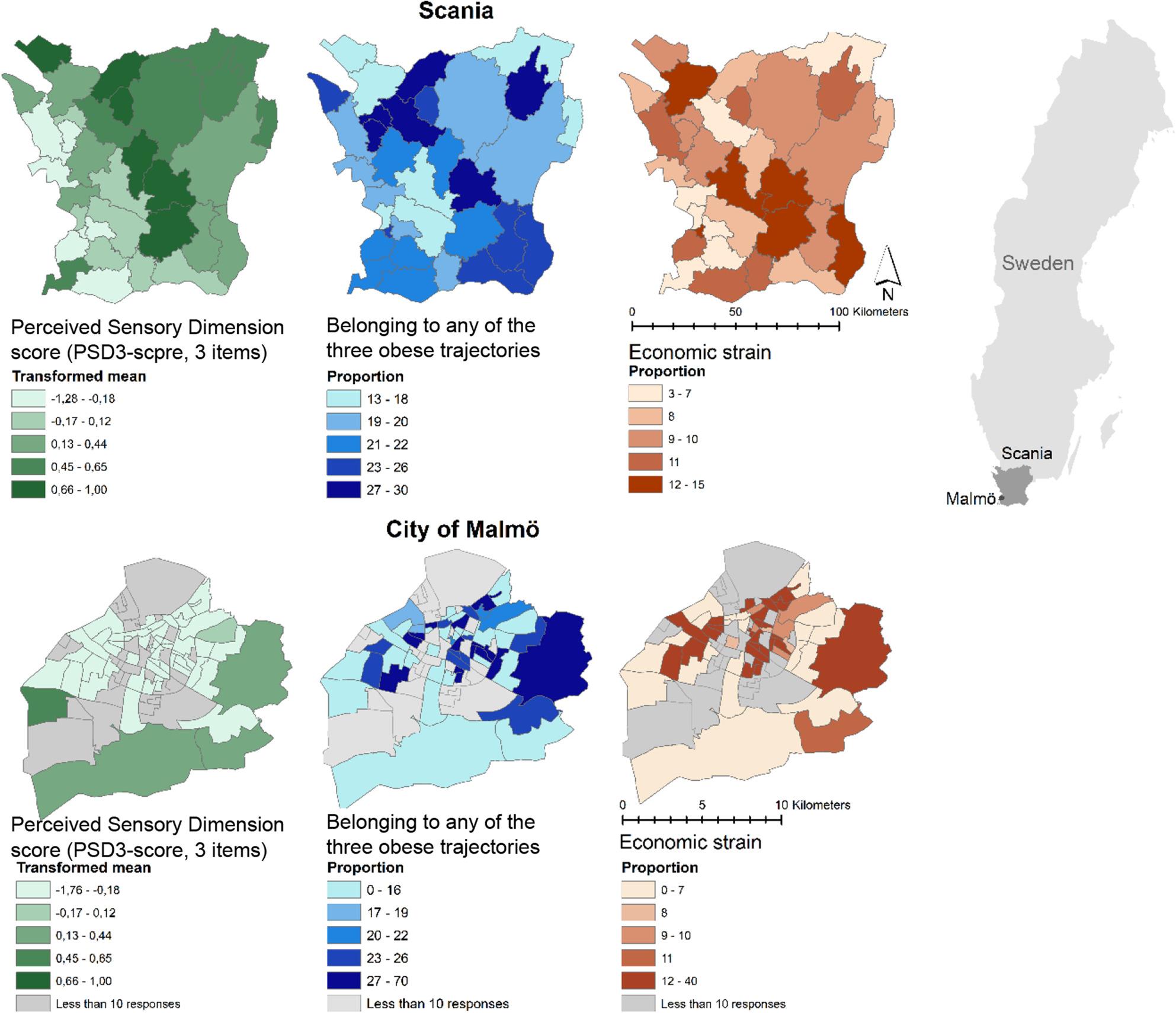
Visualization of the Perceived Sensory Dimension score (PSD3-score, 3 items), trajectory membership and economic strain across Malmö and Scania, based on the location of the dwelling in 2000, Scania Public Health Cohort, Sweden, 2000. (Proportions categorized in percentiles for Scania)

The geographical variations of the PSD3-score, trajectory membership and economic strain across municipalities in Scania and across Regional Statistical Areas (RegSo) in the city of Malmö (largest city in Scania) are mapped in Fig. 2. Educational level and other dimensions are mapped in Supplementary Figure S2. Some geographical variation could be observed by visual inspection of the PSD3-score, with the highest PSD3-score in rural areas in opposition to lowest score around the three major cities. Regarding BMI trajectories, the highest proportions of participants belonging to *obese* trajectories were found in the central and north-western areas of Scania. Moderate proportions were found around the three major cities. Highest proportions of economic strain were observed in central Scania and relatively high levels around major cities. Geographical variation was also observed in the city of Malmö, with highest PSD3-score further from the city centre and highest proportions of participants belonging to *obese* trajectories in the western parts. Proportions of participants declaring economic strain seemed to be spatially correlated to proportions of participants belonging to *obese* trajectories.

### Association with environmental characteristics

Environmental characteristics of the participants’ environment are presented in Table 2. No significant differences were found between *obese* and *non-obese* trajectories for urban/rural residency in any of the sex-age strata. The PSD3-score was not significantly different between *non-obese* and *obese* trajectories, except for women in the youngest age strata, with a lower PSD3-score in the merged *non*-*obese* trajectories.

**Table 2:** Characteristics of the participants’ dwelling and residential environment at baseline across grouped trajectories stratified by sex and age, Scania Public Health Cohort, Sweden, 2000–2016 (*N* = 10046)

	Men						Women					
	18–39 years (*N* = 1628)		40–59 years (*N* = 2007)		60 + years (*N* = 893)		18–39 years (*N* = 2140)		40–59 years (*N* = 2410)		60 + years (*N* = 968)	
Trajectories	Non- obese	Obese	Non- obese	Obese	Non- obese	Obese	Non- obese	Obese	Non- obese	Obese	Non- obese	Obese
N	1107	521	1620	387	782	111	1619	521	1993	417	876	92
Living in urban/rural area, n	999	481	1594	381	759	108	1427	462	1965	411	857	90
Living in urban/rural area, %												
Urban	87.19	85.24	83.63	83.99	84.72	85.19	87.32	84.20	84.94	84.91	87.75	85.56
Rural	12.81	14.76	16.37	16.01	15.28	14.81	12.68	15.80	15.06	15.09	12.25	14.44
Housing type, n	1077	505	1945	402	755	106	1574	500	1945	402	853	87
Housing type, %												
Villa or chain-house	52.27	55.64	78.59	70.03	76.03	76.42	54.38	52.80	74.76	68.91	69.75	73.56
Other	47.73	44.36	21.41	29.97	23.97	23.58	45.62	47.20	25.24	31.09	30.25	26.44
Housing ownership, n	1068	501	1581	376	752	108	1538	496	1946	410	848	90
Housing ownership,%												
Ownership / condominium / lease	45.60	47.70	73.43	62.23	72.07	74.07	46.36	45.16	69.06	62.44	64.15	62.22
Condominium - paid contribution to the housing legal association	15.82	16.57	10.88	14.89	14.49	12.04	15.21	15.73	13.77	14.88	18.51	15.56
Tenancy	38.58	35.73	15.69	22.87	13.43	13.89	38.43	39.11	17.16	22.68	17.33	22.22
Perceived sensory dimensions												
Perceived Sensory Dimension score (PSD3-score), 3 items, standardized, n	960	466	1520	352	720	101	1361	439	1868	390	806	85
Mean	-0.17	-0.09	0.09	0.07	0.05	0.13	-0.14	-0.02	0.09	0.07	-0.01	0.06
SD	1.01	0.99	1.00	1.00	0.98	0.98	1.01	1.05	1.00	0.95	0.96	0.87
Median	-0.44	-0.26	-0.01	-0.01	-0.06	0.09	-0.33	-0.23	-0.05	-0.10	-0.15	0.04
Neighbourhood coherence, n	979	474	1556	370	746	102	1398	450	1923	402	830	89
Mean	0.78	0.78	0.79	0.78	0.78	0.78	0.78	0.78	0.79	0.78	0.79	0.79
SD	0.03	0.03	0.03	0.03	0.03	0.03	0.03	0.03	0.03	0.03	0.03	0.03
Neighbourhood safety, n	982	476	1561	373	748	103	1403	452	1929	402	835	89
Mean	0.82	0.81	0.84	0.83	0.83	0.83	0.82	0.82	0.83	0.83	0.83	0.83
SD	0.07	0.14	0.07	0.07	0.07	0.08	0.08	0.08	0.07	0.07	0.07	0.06
Recreational possibilities, n	965	471	1512	359	733	101	1386	440	1882	391	820	87
Mean	0.82	0.81	0.82	0.81	0.82	0.81	0.83	0.81	0.82	0.80	0.84	0.83
SD	0.13	0.14	0.13	0.14	0.13	0.14	0.13	0.14	0.13	0.14	0.12	0.13

*SD* Standard Deviation

Non-obese trajectories correspond to *Stable-normal* and *stable-overweight* trajectories, Obese trajectories correspond to *Fluctuating-overweight*, *increasing-obesity* and *fluctuating-obesity* trajectories

Regarding other dimensions, no significant differences were observed for safety, while some differences in recreational possibilities could be observed in the youngest age strata for men, and in the youngest and intermediate age strata for women. Neighbourhood coherence was significantly different only in the youngest age strata, in men and women.

The results from logistic regression models comparing *obese* trajectories to *non-obese* trajectories are presented in Table 3. No statistically significant associations were found between PSD3-score and trajectory membership in most of the models. Positive associations were found between PSD3-score and *obese* trajectories among all participants and specifically among women in model 1 in the youngest age group, but these associations attenuated and lost statistical significance after covariate adjustments.

**Table 3:** Logistic regression analysis stratified by sex and age for the association between the Perceived Sensory Dimension score (PSD3-score, 3 items) assessed for the dwelling at baseline and membership in any of the three obese BMI trajectories, Scania Public Health Cohort, 2000–2016, Sweden (*N* = 10046)

**Men**	** 18–39 years**				** 40–59 years**				** 60 + years**			
	***N***	**OR**	**95% CI**	***P***	***N***	**OR**	**95% CI**	***P***	***N***	**OR**	**95% CI**	***P***
Model 1	1426	1.09	(0.97; 1.21)	0.14	1872	0.99	(0.88; 1.11)	0.81	821	1.06	(0.86; 1.32)	0.58
Model 2	1383	1.04	(0.92; 1.17)	0.52	1813	1.04	(0.92; 1.17)	0.56	766	1.08	(0.86; 1.36)	0.53
Model 3	1285	1.04	(0.91; 1.20)	0.54	1698	1.14	(0.99; 1.31)	0.07	678	1.22	(0.94; 1.59)	0.14
Model 4	1383	1.03	(0.90; 1.16)	0.71	1813	1.07	(0.94; 1.22)	0.33	766	1.15	(0.90; 1.47)	0.27
Model 5	1367	1.07	(0.92; 1.24)	0.36	1776	1.11	(0.95; 1.29)	0.19	755	1.26	(0.95; 1.68)	0.12
**Women**	**18–39 years**				**40–59 years**				** 60 + years**			
	***N***	**OR**	**95% CI**	***P***	***N***	**OR**	**95% CI**	***P***	***N***	**OR**	**95% CI**	***P***
Model 1	1800	1.12	(1.01; 1.25)	0.03	2258	0.98	(0.87; 1.09)	0.68	891	1.08	(0.85; 1.36)	0.54
Model 2	1740	1.08	(0.97; 1.21)	0.17	2164	1.01	(0.90; 1.14)	0.83	800	1.08	(0.83; 1.38)	0.59
Model 3	1581	1.12	(0.99; 1.28)	0.08	2000	1.02	(0.89; 1.16)	0.78	724	1.09	(0.81; 1.46)	0.58
Model 4	1740	1.06	(0.94; 1.19)	0.39	2164	1.01	(0.89; 1.15)	0.90	800	1.04	(0.79; 1.37)	0.77
Model 5	1720	1.14	(0.99; 1.31)	0.06	2131	0.97	(0.84; 1.12)	0.71	790	1.14	(0.84; 1.57)	0.40
**All**	** 18–39 years**				** 40–59 years**				** 60 + years**			
	***N***	**OR**	**95% CI**	***P***	***N***	**OR**	**95% CI**	***P***	***N***	**OR**	**95% CI**	***P***
Model 1	3226	1.11	(1.02; 1.19)	0.01	4130	0.98	(0.91; 1.06)	0.64	1712	1.07	(0.91, 1.25)	0.41
Model 2	3123	1.06	(0.98; 1.15)	0.14	3977	1.09	(0.94; 1.12)	0.56	1566	1.07	(0.91; 1.27)	0.42
Model 3	2866	1.09	(0.99; 1.19)	0.09	3698	1.07	(0.97; 1.17)	0.18	1402	1.14	(0.94; 1.39)	0.18
Model 4	3123	1.04	(0.95; 1.14)	0.37	3977	1.09	(0.92; 1.28)	0.41	1566	1.10	(0.92; 1.31)	0.32
Model 5	3087	1.09	(0.99; 1.21)	0.08	3907	1.04	(0.93; 1.15)	0.51	1545	1.21	(0.98; 1.49)	0.08

*OR* Odds Ratio; 95% CI: 95% Confidence Interval; P: p-value. Merged stable trajectories are taken as the reference, and the probability of belonging to the merged obese trajectories is modelled

Model 1: adjusted for age (timescale). / Model 2: adjusted for age (timescale), economic strain, education level and birth in a Nordic country. / Model 3: adjusted for model 2 + housing type and relation to employment / Model 4: adjusted for model 2 + living in an urban/rural area / Model 5: adjusted for model 2 + living in an urban/rural area + inclusion of other exposure variables (neighbourhood coherence, neighbourhood safety and recreational possibilities)

## Discussion

### Main findings

In this sample of the SPHC, five BMI trajectories were identified. Two trajectories started in the normal/overweight category and were relatively stable over time, while the three other ones were close to or above the commonly used threshold for obesity. Overall, more favourable socioeconomic and lifestyle factors were observed in *non-obese* trajectories. The PSD3-score varied geographically across Scania, but little differences were observed for the selected characteristics of the participants’ environment in relation to BMI trajectories.

### Comparison with literature

To our knowledge, the present work is the first to evaluate associations between PSDs of the environment and BMI trajectories. As a result, it is difficult to directly compare our findings to prior scientific literature.

Obesity prevalence (BMI ≥ 30 kg/m^2^) was 10% in men and 8% in women, in 2000 in our sample, similarly to the national obesity prevalence in 2000, which was 10% for both men and women [22]. When it comes to BMI trajectories, we identified five trajectories in this sample. BMI trajectories have not been previously studied in relation to environmental features during adulthood; nonetheless, previous studies identifying BMI trajectories during adulthood, mainly in the field of cancer epidemiology, have also identified 4–5 trajectories in their research [14, 23, 24].

Whilst it is difficult to compare the shapes of the trajectories from one study to another, it is possible to observe common characteristics. In Lennon et al..’s study, five trajectories were identified in both men and women [14]. The “stable normal weight” and “normal weight to overweight” trajectories comprising the largest number of participants were similar to the two stable trajectories identified in our study. In this same study, the two trajectories ending in obesity (Class IV and V), comprising less participants, also had similar shapes to the *fluctuating-obesity* and *increasing-obesity* identified in our study. Two similar stable trajectories were also found in the study by Song et al. [23].

Many studies have been conducted on the associations between greenness-related measures and BMI, or overweight and obesity, concluding in mixed results [25–30]. A systematic review and meta-analysis published in 2020 concluded that there might be an association between greater access to greenspace and lower odds of overweight/obesity, without being able to draw a robust conclusion [31]. However, these associations have been studied using heterogenous measures of greenspace, mainly objective and in cross-sectional designs [31].

Regarding the trajectory-approach, it has been used to assess the associations between the environment and overweight or obesity mainly during childhood and adolescence [30].

One of our findings was the relation between socioeconomic factors and BMI trajectories. Individuals classified in the stable trajectories had overall a more favourable socioeconomic profile than individuals classified in the obese trajectories. It is an expected result that individuals having lower socioeconomic status are more likely to become obese and this has been reported in several other studies conducted in developed countries [32]. More specifically in the context of environment and health relationships, two systematic reviews have highlighted gender and socioeconomic status as potential moderators in the greenspace-health associations [10, 33]. While there seemed to be a role of socioeconomic status in our study, we did not investigate gender differences.

### Possible mechanisms

Two main pathways linking greenspace to health are applicable when studying obesity [8, 9, 34]. Regarding the first pathway, availability and access to greenspace is thought to be encouraging physical activity and therefore preventing overweight and obesity. In our study, we have observed higher proportions of participants declaring a sedentary lifestyle in the *Obese* trajectories group, but a different design or more advanced statistical techniques would be required to study the link between PSD3-score and physical activity. The second pathway, through restoration, is more indirect in the case of metabolic diseases [35] and was not investigated in this work.

In this study, we focused on the quality of the natural outdoor environments in relation to obesity development rather than more largely “greenspace”. However, as the quality is most likely associated with quantity of natural environments close to the residence, it seems unlikely that our study would not have captured at least medium to strong associations between quantity of natural environments and obesity development. Therefore, we think the mechanisms at play would be similar to the ones observed in prior studies. Nevertheless, we cannot rule out that other aspects of the outdoor environment not included in the present study might play a role in obesity development.

### Strengths and limitations

Several strengths can be highlighted in our study. Firstly, the SPHC data allowed us to look at repeated measures of BMI, in a longitudinal design, following participants up to 16 years, from a wide range of age groups. A variety of variables was available for different timepoints, making it possible to allow for stratification and thus adjust for main confounders and study interactions. Second, the use of trajectories made it possible to capture variation over time and among groups, as opposed to a single or averaged measurements approach or a simple mixed model, which assumes that all the population follows the same trajectory. Thirdly, this study used PSDs as exposure variables, which is a novel way to characterize neighbourhoods, likely to capture significant effects on health-related behaviours [36]. While based on perceptions, these aggregated measures have shown clear associations with objective measures of the outdoor environment and might overcome single-source bias [12]. In addition, the ScOut database made it possible to study several dimensions simultaneously as well as their interactions.

There are also some limitations to this study. Firstly, body weight and height, used for BMI calculation, were self-reported. While potential outliers were excluded, measurement errors cannot be ruled out. Second, the assessments included in the ScOut database correspond to the period from 2008 to 2020. These assessments were matched to SPHC even for participants included in 2000 and 2005, leading to a time lag for the exposure between 2000 and 2008. Nevertheless, changes in the outdoor environment are slow processes and it seems unlikely that the outdoor environment has changed significantly, over this period, on a population level. Moreover, this study was conducted on a selected sample of 10 046 participants from the SPHC (*N* = 15 916), which had a 58% participation rate. A previous study had detected under-represented groups in the survey, including men, individuals with low level of education, and immigrants [15]. However, a more recent study, designed to reweight this survey, concluded that reweighting had only a limited impact, indicating that associations observed in the SPHC cohort were relatively representative of the target population [37]. Finally, while GBTM has many advantages, it also has limitations. It allows to examine differences between subgroups but does not allow to study differences within subgroups. In addition, model specification when using this type of methods is sometimes a challenging compromise between different measures of adequacy and an acceptable number of classes that include a significant proportion of the population. Other approaches may be complementary.

## Conclusion

In this study, we observed less favourable sociodemographic and lifestyle factors (smoking, sedentarity, lower education, higher economic strain) in participants belonging to obese trajectories but no consistent associations between residential environmental factors and BMI trajectories were observed. The study of overweight and obesity over time is complex and involving a wide range of factors beyond environmental ones. In addition, residential environmental factors are challenging to study in epidemiology, involving a number of interactions. However, the use of aggregated PSDs still seems promising for the study of environment and health associations, and complementary to objective measures. More studies are needed to better understand the underlying potential mechanisms linking environment, established individual-level risk factors and health outcomes before translating into public policies.

## Supplementary Information

Supplementary Material 1

## Data Availability

The ScOut database and the Scania Public Health Cohort are hosted by the Lund University Population Research Platform (LUPOP). Area-level data can be made available upon request to info-lupop@med.lu.se to interested researchers, but access to individual-level data will generally require a new ethical permission.

## Abbreviations

BIC: Bayesian Information Criterion
BMI: Body Mass Index
DeSO: Demographic Statistical Area
GBTM: Group-Based Trajectory Modelling
PSD: Perceived Sensory Dimension
PSD3-score: Perceived Sensory Dimension score (3 items)
RegSo: Regional Statistical Area
ScOut: Scania outdoor environment (database)
SD: Standard Deviation
SPHC: Scania Public Health Cohort
