# Supplementary material for: Individual and environmental determinants of body mass index trajectories: results from a longitudinal study in Southern Sweden
Source: BMC Public Health. 2026 Apr 17;26:1273. doi: 10.1186/s12889-026-27378-1 (PMC13091265; doi:10.1186/s12889-026-27378-1)
Supplement: Supplementary file 1 — Supplementary Material 1 [file 12889_2026_27378_MOESM1_ESM.docx]

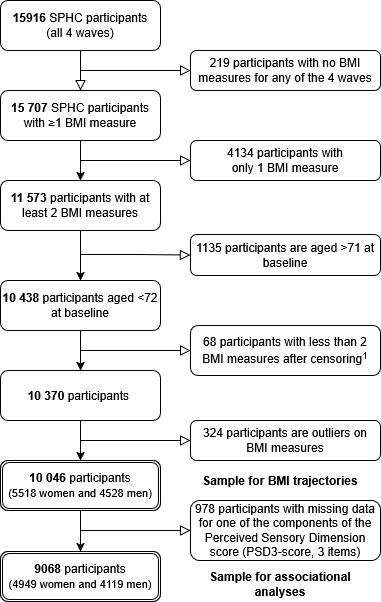


SPHC: Scania Public Health Cohort; BMI, Body Mass Index ^1^: Participants aged 72 years or older at baseline were excluded, and participants were censored during follow-up when they turned 76**.

Supplementary Figure S1: Sample selection, Scania Public Health Cohort, 2000-2016**


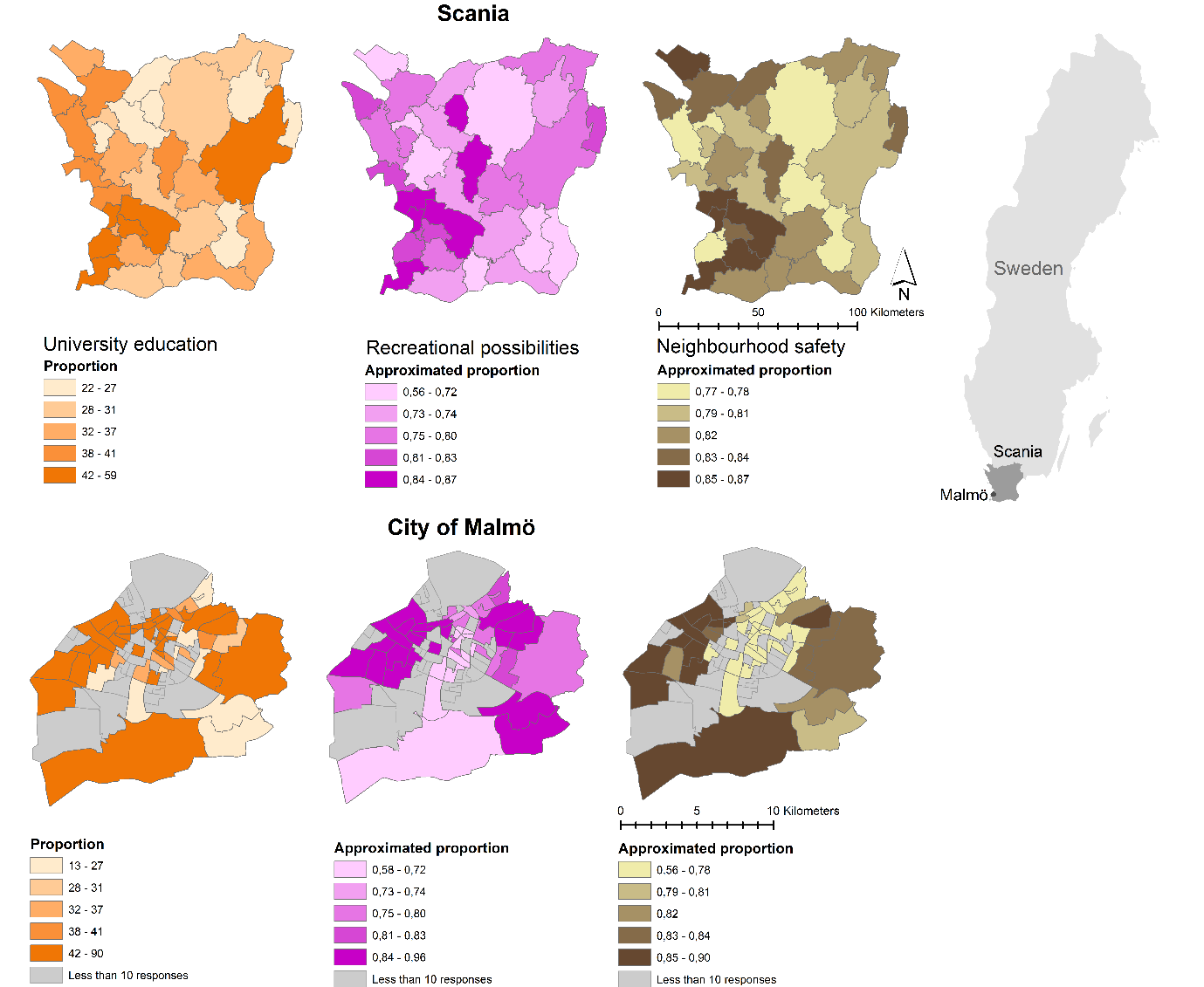


**Supplementary Figure S2**: Visualization of educational level and other perceived sensory dimensions across Malmö and Scania, based on the location of the dwelling in 2000, Scania Public Health Cohort, Sweden, 2000. (Proportions categorized in percentiles for Scania)

**Supplementary Table S1**: Criteria for model selection by gender, Scania Public Health Cohort, 2000-2016, Sweden (N=10 046)

| **Model** | **K** | **Number  of parameters** | **Bayesian Information Criterion** | **Proportions per class (%)** | | | | | | **Entropy (close to 0)** | **Relative entropy (close to 1)** | **Average Posterior Probability of Assignment (>0.7)** | | | | | | **Odds of Correct Classification (>5)** | | | | | |
| --- | --- | --- | --- | --- | --- | --- | --- | --- | --- | --- | --- | --- | --- | --- | --- | --- | --- | --- | --- | --- | --- | --- | --- |
|  |  |  |  |  |  |  |  |  |  |  |  |  |  |  |  |  |  |  |  |  |  |  |  |
|  |  |  |  | **Class** | **Class** | **Class** | **Class** | **Class** | **Class** |  |  | **Class** | **Class** | **Class** | **Class** | **Class** | **Class** | **Class** | **Class** | **Class** | **Class** | **Class** | **Class** |
|  |  |  |  | **1** | **2** | **3** | **4** | **5** | **6** |  |  | **1** | **2** | **3** | **4** | **5** | **6** | **1** | **2** | **3** | **4** | **5** | **6** |
| **Men** | 4 | 22 | 60344.94 | 74.0 | 21.4 | 2.4 | 2.3 |  |  | 2010.40 | 0.68 | 0.86 | 0.74 | 0.82 | 0.81 |  |  | 2.88 | 8.45 | 102.54 | 138.27 |  |  |
| **N=4528** | 5 | 27 | 60322.38 | 17.8 | 62.7 | 2.4 | 14.8 | 2.3 |  | 2914.47 | 0.60 | 0.75 | 0.74 | 0.82 | 0.68 | 0.81 |  | 11.29 | 2.47 | 95.09 | 9.32 | 145.50 |  |
|  | 6 | 32 | 60301.75 | 10.5 | 29.3 | 46.6 | 9.3 | 2.3 | 1.9 | 3493.88 | 0.57 | 0.72 | 0.67 | 0.67 | 0.70 | 0.81 | 0.80 | 17.39 | 4.58 | 3.29 | 18.37 | 95.20 | 170.24 |
| **Women** | 4 | 22 | 78043.08 | 6.0 | 26.0 | 59.6 | 8.4 |  |  | 3632.60 | 0.53 | 0.70 | 0.64 | 0.79 | 0.79 |  |  | 15.42 | 4.76 | 3.50 | 37.76 |  |  |
| **N=5518** | 5 | 27 | 77992.18 | 5.5 | 43.8 | 9.3 | 37.5 | 3.9 |  | 4164.71 | 0.53 | 0.71 | 0.67 | 0.61 | 0.72 | 0.72 | 0.80 | 18.40 | 3.14 | 14.31 | 5.01 | 81.38 |  |
|  | 6 | 32 | 78004.41 | 38.1 | 42.6 | 4.6 | 9.6 | 1.1 | 4.0 | 4502.67 | 0.55 | 0.72 | 0.65 | 0.66 | 0.62 | 0.72 | 0.80 | 4.80 | 303 | 20.04 | 14.07 | 75.20 | 77.68 |

*Recommended values for each criterion are indicated in brackets.*

**Supplementary Table S2**: Baseline characteristics of the participants, Scania Public Health Cohort 2000-2016 (N=10046)

|  | **Total (N=10 046)** | **Men (N=4528)** | **Women (N=5518)** |
| --- | --- | --- | --- |
| **Age (years) at baseline**, Mean (SD) | 44.93 (14.26) | 45.51 (14.25) | 44.5 (14.3) |
| **BMI (kg/m^2^) at baseline**, Mean (SD) | 24.82 (3.71) | 25.55 (3.40) | 24.23 (3.85) |
| **Inclusion year, %** |  |  |  |
| 2000 | 95.33 | 96.51 | 94.36 |
| 2005 | 1.44 | 0.93 | 1.87 |
| 2010 | 3.23 | 2.56 | 3.77 |
| **Economic strain, %** |  |  |  |
| **N** | 9840 | 4449 | 5391 |
| No | 90.36 | 91.41 | 89.48 |
| Yes | 9.64 | 8.59 | 10.52 |
| **Born in a nordic country, %** |  |  |  |
| **N** | 9966 | 4500 | 5466 |
| No | 7.82 | 8.09 | 7.59 |
| Yes | 92.18 | 91.91 | 92.41 |
| **Education level, %** |  |  |  |
| **N** | 9760 | 4423 | 5337 |
| Primary | 25.67 | 27.40 | 24.23 |
| Secondary | 32.97 | 32.60 | 33.28 |
| university/college | 41.36 | 40.00 | 42.50 |
| **Marital status, %** |  |  |  |
| **N** | 9902 | 4469 | 5433 |
| Married/sambo | 72.26 | 72.48 | 72.08 |
| Unmarried | 18.25 | 20.14 | 16.69 |
| Divorced | 7.33 | 6.33 | 8.15 |
| Widow, widower | 2.16 | 1.05 | 3.07 |
| **Physical activity, %** |  |  |  |
| **N** | 9788 | 4420 | 5368 |
| Sedentary | 14.00 | 14.75 | 13.38 |
| Moderate exercise | 61.28 | 59.66 | 62.61 |
| Regular exercise | 21.74 | 20.68 | 22.62 |
| Hard-training/competitive exercise | 2.98 | 4.91 | 1.40 |
| **General Health, %** |  |  |  |
| **N** | 10011 | 4514 | 5497 |
| Very good | 21.10 | 22.80 | 19.70 |
| Good | 52.41 | 53.30 | 51.68 |
| Fairly good | 21.36 | 19.58 | 22.81 |
| Bad | 4.44 | 3.74 | 5.00 |
| Very bad | 0.70 | 0.58 | 0.80 |
| **Long-term illness/Disability, %** |  |  |  |
| **N** | 9444 | 4283 | 5161 |
| Yes | 27.73 | 26.73 | 28.56 |
| No | 72.27 | 73.27 | 71.44 |
| **Smoking habits, %** |  |  |  |
| **N** | 9816 | 4430 | 5386 |
| Never smoker | 49.89 | 48.47 | 51.06 |
| Past smoker | 29.43 | 32.84 | 26.62 |
| Current smoker | 20.68 | 18.69 | 22.32 |
| **Alcohol consumption, %** |  |  |  |
| **N** | 9479 | 4319 | 5160 |
| not in the last year | 7.84 | 6.07 | 9.32 |
| not in the last month | 9.21 | 6.37 | 11.59 |
| less than 10 days out of 30 | 74.20 | 75.32 | 73.26 |
| 10-20 days out of 30 | 6.44 | 8.68 | 4.55 |
| 20-30 days out of 30 | 2.32 | 3.57 | 1.28 |
| **Housing ownership, %** |  |  |  |
| **N** | 9714 | 4386 | 5328 |
| Ownership/condominium/lease | 60.53 | 62.54 | 58.88 |
| Condominium - contribution to housing association | 14.56 | 13.73 | 15.24 |
| Tenancy | 24.91 | 23.73 | 25.88 |
| **Living in urban/rural area, %** |  |  |  |
| **N** | 9534 | 4322 | 5212 |
| Rural | 14.51 | 15.11 | 14.01 |
| Urban | 85.49 | 84.89 | 85.99 |
| **Feeling rooted in residential area, %** |  |  |  |
| **N** | 9851 | 4425 | 5426 |
| Highly | 38.78 | 38.51 | 39.00 |
| To some extent | 36.66 | 36.02 | 37.17 |
| Not especially | 19.30 | 19.86 | 18.84 |
| Not at all | 5.27 | 5.60 | 4.99 |

**Supplementary Table S3**: Baseline sociodemographic characteristics of men participants across BMI trajectories and stratified by age, Scania Public Health Cohort, Sweden, 2000-2016 (N=4528)

|  |  |  |  |  |  |  |  | **18-39 years old (N=1628)** | | | | | **40-59 years old (N=2007)** | | | | | **60+ years old (N=893)** | | | | |
| --- | --- | --- | --- | --- | --- | --- | --- | --- | --- | --- | --- | --- | --- | --- | --- | --- | --- | --- | --- | --- | --- | --- |
| **Trajectories** | **All** | **SN** | **SO** | **IO** | **FOv** | **FOb** | p-value^1^ | **SN** | **SO** | **IO** | **FOv** | **FOb** | **SN** | **SO** | **IO** | **FOv** | **FOb** | **SN** | **SO** | **IO** | **FOv** | **FOb** |
| N | **4528** | 669 | 2840 | 110 | 806 | 103 |  | 197 | 910 | 39 | 413 | 69 | 352 | 1268 | 56 | 307 | 24 | 120 | 662 | 15 | 86 | 10 |
| % of sample |  | 14.8 | 62.7 | 2.4 | 17.8 | 2.3 |  |  |  |  |  |  |  |  |  |  |  |  |  |  |  |  |
| **Age (years) at baseline, Mean (SD)** | 45.5 | 46.7 | 46.9 | 42.9 | 41.0 | 36.8 |  | 30.0 | 29.3 | 27.0 | 29.8 | 29.0 | 50.3 | 50.3 | 48.6 | 49.7 | 48.1 | 64.4 | 64.6 | 63.0 | 63.8 | 63.8 |
|  | (14.3) | (13.4) | (14.4) | (13.8) | (13.4) | (13.4) |  | (6.4) | (6.2) | (5.9) | (5.9) | (6.3) | (5.4) | (5.5) | (5.5) | (5.7) | (6.3) | (3.2) | (3.2) | (2.3) | (2.9) | (3.3) |
| **BMI (kg/m^2^) at baseline, Mean (SD)** | 25.6 | 21.6 | 25.3 | 28.9 | 28.8 | 31.3 |  | 20.8 | 23.9 | 30.8 | 26.9 | 30.4 | 21.9 | 25.7 | 27.4 | 30.3 | 34.0 | 21.8 | 26.2 | 29.8 | 32.1 | 30.7 |
|  | (3.4) | (1.3) | (2.1) | (5.2) | (3.4) | (5.3) |  | (1.4) | (1.9) | (5.2) | (3.1) | (5.1) | (1.17) | (1.7) | (4.9) | (2.1) | (5.0) | (1.1) | (2.1) | (4.9) | (3.0) | (5.5) |
| **Economic strain, %** |  |  |  |  |  |  | <0.0001 |  |  |  |  |  |  |  |  |  |  |  |  |  |  |  |
| **N** | 4449 | 656 | 2792 | 107 | 793 | 101 |  | 189 | 897 | 38 | 408 | 68 | 351 | 1249 | 54 | 303 | 23 | 116 | 646 | 15 | 82 | 10 |
| No | 91.4 | 92.5 | 92.9 | 85.1 | 87.6 | 79.2 |  | 89.4 | 90.3 | 81.6 | 86.5 | 79.4 | 92.6 | 93.0 | 83.3 | 88.1 | 73.9 | 97.4 | 96.3 | 100 | 91.5 | 90.0 |
| Yes | 8.6 | 7.5 | 7.1 | 15.0 | 12.4 | 20.8 |  | 10.6 | 9.7 | 18.4 | 13.5 | 20.6 | 7.4 | 7.0 | 16.7 | 11.9 | 26.1 | 2.6 | 3.7 | 0.0 | 8.5 | 10.0 |
| **Born in a Nordic country, %** |  |  |  |  |  |  | 0.26 |  |  |  |  |  |  |  |  |  |  |  |  |  |  |  |
| **N** | 4500 | 668 | 2820 | 109 | 800 | 103 |  | 196 | 906 | 39 | 411 | 69 | 352 | 1255 | 55 | 304 | 24 | 120 | 659 | 15 | 85 | 10 |
| No | 8.1 | 6.7 | 7.9 | 8.3 | 9.9 | 8.7 |  | 9.7 | 7.8 | 5.1 | 11.0 | 7.3 | 5.7 | 8.7 | 12.7 | 9.2 | 12.5 | 5.0 | 6.4 | 0.0 | 7.1 | 10.0 |
| Yes | 91.9 | 93.3 | 92.1 | 91.7 | 90.1 | 91.3 |  | 90.3 | 92.2 | 94.9 | 89.1 | 92.8 | 94.3 | 91.3 | 87.3 | 90.8 | 87.5 | 95.0 | 93.6 | 100.0 | 92.9 | 90.0 |
| **Education level, %** |  |  |  |  |  |  | <0.0001 |  |  |  |  |  |  |  |  |  |  |  |  |  |  |  |
| **N** | 4423 | 659 | 2774 | 108 | 781 | 101 |  | 193 | 899 | 38 | 405 | 68 | 349 | 1244 | 55 | 295 | 24 | 117 | 631 | 15 | 81 | 9 |
| primary | 27.4 | 25.0 | 28.3 | 30.6 | 26.3 | 22.8 |  | 3.1 | 4.6 | 10.5 | 9.6 | 8.8 | 27.2 | 31.5 | 30.9 | 38.6 | 45.8 | 54.7 | 55.9 | 80.0 | 64.2 | 66.7 |
| secondary | 32.6 | 28.5 | 30.4 | 36.1 | 42.4 | 41.6 |  | 44.6 | 49.7 | 47.4 | 57.5 | 52.9 | 23.5 | 25.7 | 36.4 | 29.5 | 16.7 | 17.1 | 11.9 | 6.7 | 13.6 | 22.2 |
| university/college | 40.0 | 46.4 | 41.3 | 33.3 | 31.4 | 35.6 |  | 52.3 | 45.7 | 42.1 | 32.8 | 38.2 | 49.3 | 42.8 | 32.7 | 31.9 | 37.5 | 28.2 | 32.2 | 13.3 | 22.2 | 11.1 |
| **Physical activity, %** |  |  |  |  |  |  | <0.0001 |  |  |  |  |  |  |  |  |  |  |  |  |  |  |  |
| **N** | 4420 | 656 | 2769 | 108 | 789 | 98 |  | 195 | 896 | 39 | 407 | 67 | 344 | 1237 | 55 | 299 | 21 | 117 | 636 | 14 | 83 | 10 |
| Sedentary | 14.8 | 14.2 | 11.8 | 23.2 | 21.3 | 39.8 |  | 22.1 | 11.8 | 25.6 | 21.9 | 35.8 | 10.8 | 13.4 | 21.8 | 21.1 | 57.1 | 11.1 | 8.7 | 21.4 | 19.3 | 30.0 |
| Moderate exercise | 59.7 | 59.5 | 60.9 | 61.1 | 57.3 | 42.9 |  | 41.5 | 44.1 | 53.9 | 47.7 | 40.3 | 65.4 | 64.8 | 63.6 | 65.2 | 38.1 | 71.8 | 77.2 | 71.4 | 75.9 | 70.0 |
| Regular exercise | 20.7 | 23.6 | 22.0 | 13.9 | 15.6 | 12.2 |  | 31.8 | 29.9 | 18.0 | 19.7 | 16.4 | 21.2 | 20.5 | 12.7 | 13.0 | 4.8 | 17.1 | 13.8 | 7.1 | 4.8 | 0.0 |
| Hard-training/competitive | 4.9 | 2.7 | 5.3 | 1.9 | 5.8 | 5.1 |  | 4.6 | 14.2 | 2.6 | 10.8 | 7.5 | 2.6 | 1.4 | 1.8 | 0.7 | 0.0 | 0.0 | 0.3 | 0.0 | 0.0 | 0.0 |
| **General Health, %** |  |  |  |  |  |  | <0.0001 |  |  |  |  |  |  |  |  |  |  |  |  |  |  |  |
| **N** | 4514 | 667 | 2832 | 110 | 802 | 103 |  | 197 | 908 | 39 | 412 | 69 | 350 | 1263 | 56 | 305 | 24 | 120 | 661 | 15 | 85 | 10 |
| Very good | 22.8 | 24.3 | 23.8 | 15.5 | 20.1 | 13.6 |  | 26.4 | 30.8 | 10.3 | 24.8 | 15.9 | 24.0 | 22.0 | 17.9 | 17.7 | 12.5 | 21.7 | 17.7 | 20.0 | 5.9 | 0.0 |
| Good | 53.3 | 52.8 | 54.1 | 52.7 | 51.5 | 49.5 |  | 55.8 | 54.4 | 66.7 | 53.2 | 53.6 | 51.7 | 52.8 | 50.0 | 50.8 | 45.8 | 50.8 | 56.1 | 26.7 | 45.9 | 30.0 |
| Fairly good | 19.6 | 17.5 | 18.7 | 21.8 | 22.9 | 28.2 |  | 13.7 | 13.0 | 18.0 | 18.0 | 24.6 | 18.3 | 20.7 | 19.6 | 25.3 | 29.2 | 21.7 | 22.7 | 40.0 | 38.8 | 50.0 |
| Bad | 3.8 | 4.4 | 2.9 | 8.2 | 5.1 | 6.8 |  | 3.1 | 1.4 | 2.6 | 3.6 | 4.4 | 4.9 | 3.8 | 10.7 | 5.9 | 8.3 | 5.0 | 3.3 | 13.3 | 9.4 | 20.0 |
| Very bad | 0.6 | 1.1 | 0.4 | 1.8 | 0.4 | 1.9 |  | 1.0 | 0.3 | 2.6 | 0.5 | 1.5 | 1.1 | 0.6 | 1.8 | 0.3 | 4.2 | 0.8 | 0.2 | 0.0 | 0.0 | 0.0 |
| **Long-term illness/Disability, %** |  |  |  |  |  |  | 0.02 |  |  |  |  |  |  |  |  |  |  |  |  |  |  |  |
| **N** | 4283 | 637 | 2683 | 103 | 762 | 98 |  | 192 | 875 | 39 | 399 | 66 | 340 | 1228 | 54 | 292 | 22 | 105 | 580 | 10 | 71 | 10 |
| Yes | 26.7 | 25.6 | 25.5 | 34.0 | 29.9 | 34.7 |  | 22.9 | 20.1 | 33.3 | 25.1 | 28.8 | 27.7 | 25.7 | 37.0 | 32.5 | 36.4 | 23.8 | 33.5 | 20.0 | 46.5 | 70.0 |
| No | 73.3 | 74.4 | 74.5 | 66.0 | 70.1 | 65.3 |  | 77.1 | 79.9 | 66.7 | 74.9 | 71.2 | 72.4 | 74.4 | 63.0 | 67.5 | 63.6 | 76.2 | 66.6 | 80.0 | 53.5 | 30.0 |
| **Smoking habits, %** |  |  |  |  |  |  | <0.0001 |  |  |  |  |  |  |  |  |  |  |  |  |  |  |  |
| **N** | 4430 | 664 | 2771 | 109 | 784 | 102 |  | 196 | 890 | 39 | 400 | 68 | 349 | 1242 | 56 | 300 | 24 | 119 | 639 | 14 | 84 | 10 |
| Never smoker | 48.5 | 51.1 | 48.4 | 28.4 | 48.5 | 54.9 |  | 68.9 | 68.5 | 53.9 | 63.0 | 63.2 | 45.0 | 38.3 | 12.5 | 32.3 | 45.8 | 39.5 | 39.9 | 21.4 | 36.9 | 20.0 |
| Past smoker | 32.8 | 27.0 | 34.1 | 33.9 | 34.8 | 20.6 |  | 13.8 | 16.7 | 15.4 | 22.5 | 19.1 | 31.0 | 41.0 | 41.1 | 44.7 | 20.8 | 37.0 | 44.9 | 57.1 | 58.3 | 30.0 |
| Current smoker | 18.7 | 22.0 | 17.5 | 37.6 | 16.7 | 24.5 |  | 17.4 | 14.7 | 30.8 | 14.5 | 17.7 | 24.1 | 20.7 | 46.4 | 23.0 | 33.3 | 23.5 | 15.2 | 21.4 | 4.8 | 50.0 |
| **Living in urban/rural area, %** |  |  |  |  |  |  | 0.99 |  |  |  |  |  |  |  |  |  |  |  |  |  |  |  |
| **N** | 4322 | 644 | 2708 | 104 | 772 | 94 |  | 183 | 816 | 34 | 384 | 63 | 344 | 1250 | 56 | 304 | 21 | 117 | 642 | 14 | 84 | 10 |
| Urban | 84.9 | 84.6 | 85.0 | 84.6 | 84.7 | 85.1 |  | 85.8 | 87.5 | 88.2 | 84.1 | 90.5 | 81.7 | 84.2 | 83.9 | 84.9 | 71.4 | 91.5 | 83.5 | 78.6 | 86.9 | 80.0 |
| Rural | 15.1 | 15.4 | 15.0 | 15.4 | 15.3 | 14.9 |  | 14.2 | 12.5 | 11.8 | 15.9 | 9.5 | 18.3 | 15.8 | 16.1 | 15.1 | 28.6 | 8.6 | 16.5 | 21.4 | 13.1 | 20.0 |
| **Feeling rooted in residential area, %** |  |  |  |  |  |  |  |  |  |  |  |  |  |  |  |  |  |  |  |  |  |  |
| **N** | 4425 | 654 | 2773 | 107 | 791 | 100 |  | 192 | 890 | 38 | 404 | 67 | 347 | 1238 | 55 | 303 | 24 | 115 | 645 | 14 | 84 | 9 |
| Highly | 38.5 | 35.8 | 39.2 | 41.1 | 38.3 | 37.0 |  | 22.9 | 28.3 | 36.8 | 29.0 | 25.4 | 41.5 | 42.6 | 36.4 | 47.9 | 58.3 | 40.0 | 47.6 | 71.4 | 48.8 | 66.7 |
| To some extent | 36.0 | 36.4 | 36.2 | 31.8 | 35.5 | 36.0 |  | 28.7 | 35.6 | 29.0 | 37.4 | 43.3 | 38.6 | 36.2 | 38.2 | 34.3 | 25.0 | 42.6 | 37.2 | 14.3 | 31.0 | 11.1 |
| Not especially | 19.9 | 22.5 | 19.6 | 19.6 | 19.3 | 16.0 |  | 37.5 | 27.9 | 21.1 | 25.3 | 17.9 | 16.7 | 17.5 | 23.6 | 12.2 | 8.3 | 14.8 | 11.9 | 0.0 | 16.7 | 22.2 |
| Not at all | 5.6 | 5.4 | 5.1 | 7.5 | 6.8 | 11.0 |  | 10.9 | 8.2 | 13.2 | 8.4 | 13.4 | 3.2 | 3.7 | 1.8 | 5.6 | 8.3 | 2.6 | 3.3 | 14.3 | 3.6 | 0.0 |
| **Perceived Sensory Dimension score  (PSD3-score, 3 items), standardized, N** | 4119 | 608 | 2592 | 95 | 734 | 90 |  | 171 | 789 | 34 | 370 | 62 | 325 | 1195 | 49 | 284 | 19 | 112 | 608 | 12 | 80 | 9 |
| **Perceived Sensory Dimension score  (PSD3-score, 3 items), standardized, Mean** | 0 | -0.01 | 0.004 | -0.19 | 0.03 | -0.11 | 0.23 | -0.29 | -0.15 | -0.21 | -0.05 | -0.25 | 0.09 | 0.09 | -0.22 | 0.12 | 0.06 | 0.12 | 0.04 | -0.02 | 0.11 | 0.47 |
| SD | 1 | 1.02 | 1.00 | 1.00 | 1.00 | 0.96 |  | 1.04 | 1.00 | 0.97 | 1.00 | 0.93 | 1.00 | 1.00 | 0.99 | 1.00 | 1.03 | 0.98 | 0.98 | 1.24 | 0.95 | 0.88 |
| Median | 0.2 | -0.21 | -0.18 | -0.42 | -0.10 | -0.35 |  | -0.64 | -0.38 | -0.40 | -0.24 | -0.42 | -0.09 | -0.01 | -0.44 | 0.04 | 0.19 | 0.21 | -0.09 | -0.31 | 0.07 | 0.91 |

^1^p-value for comparison between classes using Chi-square test or linear regression as appropriate, excluding missing data.
SN: *Stable-normal*; SO: *Stable-overweight;* IO*: Increasing-obesity*; FOv: *Fluctuating-overweight;* FOb *Fluctuating-obesity*

**Supplementary Table S4:** Baseline sociodemographic characteristics of women participants across BMI trajectories and stratified by age, Scania Public Health Cohort, Sweden, 2000-2016 (N=5518)

|  |  |  |  |  |  |  |  | **18-39 years  (N=2140)** | | | | | **40-59 years  (N=2410)** | | | | | **60+ years  (N=968)** | | | | |  |
| --- | --- | --- | --- | --- | --- | --- | --- | --- | --- | --- | --- | --- | --- | --- | --- | --- | --- | --- | --- | --- | --- | --- | --- |
| **Trajectories** | **All** | **SN** | **SO** | **IO** | **FOv** | **FOb** | p-value^1^ | **SN** | **SO** | **IO** | **FOv** | **FOb** | **SN** | **SO** | **IO** | **FOv** | **FOb** | **SN** | **SO** | **IO** | **FOv** | **FOb** | |
| **N** | **5518** | 2072 | 2416 | 303 | 513 | 214 |  | 707 | 912 | 124 | 260 | 137 | 955 | 1038 | 152 | 212 | 53 | 410 | 466 | 27 | 41 | 24 | |
| **% of sample** |  | 37.5 | 43.8 | 5.5 | 9.3 | 3.9 |  |  |  |  |  |  |  |  |  |  |  |  |  |  |  |  | |
| **Age** (years) at T1, Mean (SD) | 44.5 | 45.8 | 45.0 | 42.9 | 40.6 | 37.3 |  | 29.1 | 29.5 | 29.8 | 29.9 | 28.0 | 50.1 | 49.6 | 49.8 | 49.1 | 48.4 | 64.7 | 64.9 | 63.8 | 64.5 | 65.6 | |
|  | (14.3) | (14.3) | (14.3) | (12.9) | (12.9) | (14.4) |  | (6.2) | (6.0) | (5.8) | (5.9) | (6.0) | (5.5) | (5.6) | (6.0) | (5.9) | (5.7) | (3.3) | (3.1) | (3.3) | (2.9) | (2.9) | |
| **BMI** (kg/m^2^) at T1, Mean (SD) | 24.2 | 21.2 | 24.8 | 28.5 | 28.4 | 30.4 |  | 20.3 | 23.2 | 28.4 | 26.3 | 28.2 | 21.4 | 25.3 | 27.9 | 30.4 | 35.6 | 22.5 | 27.1 | 32.6 | 31.0 | 31.8 | |
|  | (3.9) | (1.7) | (2.4) | (4.6) | (3.4) | (5.7) |  | (1.5) | (1.9) | (4.3) | (2.9) | (4.8) | (1.5) | (1.8) | (4.7) | (2.4) | (4.6) | (1.6) | (2.0) | (3.7) | (3.4) | (5.5) | |
| **Economic strain, %** |  |  |  |  |  |  | <.0001 |  |  |  |  |  |  |  |  |  |  |  |  |  |  |  | |
| **N** | 5391 | 2021 | 2365 | 298 | 499 | 208 |  | 692 | 895 | 122 | 254 | 132 | 936 | 1026 | 150 | 206 | 52 | 393 | 444 | 26 | 39 | 24 | |
| No | 89.5 | 91.8 | 90.7 | 80.9 | 83.8 | 79.3 |  | 87.9 | 86.4 | 79.5 | 84.7 | 78.8 | 92.8 | 91.8 | 80.7 | 80.1 | 75.0 | 96.2 | 96.9 | 88.5 | 97.4 | 91.7 | |
| Yes | 10.5 | 8.2 | 9.3 | 19.1 | 16.2 | 20.7 |  | 12.1 | 13.6 | 20.5 | 15.4 | 21.2 | 7.2 | 8.2 | 19.3 | 19.9 | 25.0 | 3.8 | 3.2 | 11.5 | 2.6 | 8.3 | |
| **Born in a Nordic country, %** |  |  |  |  |  |  | 0.01 |  |  |  |  |  |  |  |  |  |  |  |  |  |  |  | |
| **N** | 5466 | 2055 | 2387 | 302 | 511 | 211 |  | 704 | 902 | 123 | 260 | 135 | 945 | 1027 | 152 | 210 | 53 | 406 | 458 | 27 | 41 | 23 | |
| No | 7.6 | 6.4 | 8.1 | 9.6 | 9.8 | 4.7 |  | 8.8 | 9.2 | 6.5 | 8.1 | 4.4 | 5.3 | 8.3 | 12.5 | 13.8 | 5.7 | 4.9 | 5.7 | 7.4 | 0.0 | 4.4 | |
| Yes | 92.4 | 93.6 | 91.9 | 90.4 | 90.2 | 95.3 |  | 91.2 | 90.8 | 93.5 | 91.9 | 95.6 | 94.7 | 91.7 | 87.5 | 86.2 | 94.34 | 95.1 | 94.3 | 92.6 | 100.0 | 95.7 | |
| **Education level, %** |  |  |  |  |  |  | <.0001 |  |  |  |  |  |  |  |  |  |  |  |  |  |  |  | |
| **N** | 5337 | 2014 | 2326 | 297 | 499 | 201 |  | 697 | 890 | 122 | 256 | 132 | 926 | 1004 | 150 | 206 | 51 | 391 | 432 | 25 | 37 | 18 | |
| primary | 24.2 | 23.8 | 25.6 | 23.9 | 21.2 | 20.4 |  | 4.3 | 5.2 | 4.9 | 5.1 | 9.9 | 23.7 | 27.6 | 31.3 | 34.5 | 33.3 | 59.1 | 63.0 | 72.0 | 59.5 | 61.1 | |
| secondary | 33.3 | 28.1 | 34.0 | 40.4 | 43.7 | 40.8 |  | 41.9 | 45.7 | 55.7 | 53.5 | 53.0 | 23.7 | 31.0 | 32.7 | 35.4 | 21.6 | 13.9 | 16.9 | 12.0 | 21.6 | 5.6 | |
| university/college | 42.5 | 48.1 | 40.4 | 35.7 | 35.1 | 38.8 |  | 53.8 | 49.1 | 39.3 | 41.4 | 37.1 | 52.7 | 41.4 | 36.0 | 30.1 | 45.1 | 27.1 | 20.1 | 16.0 | 18.9 | 33.3 | |
| **Physical activity, %** |  |  |  |  |  |  | <.0001 |  |  |  |  |  |  |  |  |  |  |  |  |  |  |  | |
| **N** | 5368 | 2023 | 2337 | 297 | 503 | 208 |  | 698 | 894 | 122 | 256 | 135 | 926 | 1000 | 149 | 207 | 53 | 399 | 443 | 26 | 40 | 20 | |
| Sedentary | 13.4 | 10.4 | 12.8 | 20.9 | 17.9 | 27.4 |  | 13.5 | 12.8 | 17.2 | 14.1 | 21.5 | 10.2 | 13.4 | 23.5 | 22.2 | 37.7 | 5.5 | 11.5 | 23.1 | 20.0 | 40.0 | |
| Moderate exercise | 62.6 | 59.9 | 64.7 | 65.7 | 66.0 | 53.4 |  | 51.2 | 53.9 | 67.2 | 62.1 | 52.6 | 59.8 | 68.1 | 63.8 | 69.1 | 54.7 | 75.2 | 78.8 | 69.2 | 75.0 | 55.0 | |
| Regular exercise | 22.6 | 28.2 | 21.2 | 12.5 | 14.9 | 17.8 |  | 32.0 | 30.0 | 14.8 | 21.5 | 23.7 | 29.3 | 18.5 | 11.4 | 8.7 | 7.55 | 19.1 | 9.5 | 7.7 | 5.0 | 5.0 | |
| Hard-training/competitive | 1.4 | 1.6 | 1.3 | 1.0 | 1.2 | 1.4 |  | 3.4 | 3.4 | 0.8 | 2.3 | 2.2 | 0.8 | 0.0 | 1.3 | 0.0 | 0.0 | 0.3 | 0.2 | 0.0 | 0.0 | 0.0 | |
| **General Health, %** |  |  |  |  |  |  | <.0001 |  |  |  |  |  |  |  |  |  |  |  |  |  |  |  | |
| **N** | 5497 | 2060 | 2409 | 303 | 511 | 214 |  | 707 | 910 | 124 | 258 | 137 | 945 | 1034 | 152 | 212 | 53 | 408 | 465 | 27 | 41 | 24 | |
| Very good | 19.7 | 23.5 | 18.9 | 13.2 | 14.1 | 15.9 |  | 28.3 | 25.0 | 20.2 | 14.7 | 18.3 | 21.8 | 15.6 | 7.2 | 13.7 | 11.3 | 18.9 | 14.2 | 14.8 | 12.2 | 12.5 | |
| Good | 51.7 | 51.1 | 53.4 | 46.5 | 52.6 | 42.5 |  | 51.8 | 56.0 | 45.2 | 58.1 | 46.7 | 51.5 | 51.6 | 48.0 | 45.8 | 34.0 | 49.0 | 52.5 | 44.4 | 53.7 | 37.5 | |
| Fairly good | 22.8 | 20.6 | 22.3 | 30.4 | 25.4 | 32.2 |  | 15.3 | 15.3 | 27.4 | 22.1 | 27.7 | 21.3 | 26.0 | 31.6 | 29.3 | 39.6 | 28.4 | 28.0 | 37.0 | 26.8 | 41.7 | |
| Bad | 5.0 | 4.3 | 4.6 | 8.3 | 6.5 | 8.9 |  | 4.2 | 3.0 | 6.5 | 4.3 | 6.6 | 4.7 | 6.1 | 10.5 | 9.0 | 15.1 | 3.4 | 4.3 | 3.7 | 7.3 | 8.3 | |
| Very bad | 0.8 | 0.5 | 0.8 | 1.7 | 1.4 | 0.5 |  | 0.4 | 0.8 | 0.8 | 0.8 | 0.7 | 0.7 | 0.8 | 2.6 | 2.4 | 0.0 | 0.3 | 1.1 | 0.0 | 0.0 | 0.0 | |
| **Long-term illness/Disability, %** |  |  |  |  |  |  | <.0001 |  |  |  |  |  |  |  |  |  |  |  |  |  |  |  | |
| **N** | 5161 | 1954 | 2233 | 285 | 487 | 202 |  | 684 | 882 | 122 | 253 | 134 | 924 | 975 | 138 | 200 | 48 | 346 | 376 | 25 | 34 | 20 | |
| Yes | 28.6 | 26.1 | 28.3 | 36.1 | 30.0 | 42.1 |  | 21.2 | 21.8 | 28.7 | 22.5 | 35.8 | 26.0 | 29.4 | 42.8 | 36.5 | 54.2 | 35.8 | 40.4 | 36.0 | 47.1 | 55.0 | |
| No | 71.4 | 74.0 | 71.7 | 63.9 | 70.0 | 57.9 |  | 78.8 | 78.2 | 71.3 | 77.5 | 64.2 | 74.0 | 70.6 | 57.3 | 63.5 | 45.8 | 64.2 | 59.6 | 64.0 | 52.9 | 45.0 | |
| **Smoking habits, %** |  |  |  |  |  |  | 0.006 |  |  |  |  |  |  |  |  |  |  |  |  |  |  |  | |
| **N** | 5386 | 2020 | 2356 | 301 | 499 | 210 |  | 689 | 892 | 123 | 255 | 135 | 936 | 1015 | 152 | 206 | 52 | 395 | 449 | 26 | 38 | 23 | |
| Never smoker | 51.1 | 52.7 | 51.1 | 43.2 | 51.5 | 45.2 |  | 63.7 | 59.9 | 56.1 | 60.4 | 45.2 | 44.3 | 39.7 | 33.6 | 41.8 | 36.5 | 53.4 | 59.2 | 38.5 | 44.7 | 65.2 | |
| Past smoker | 26.6 | 25.5 | 27.3 | 26.9 | 27.9 | 25.7 |  | 17.6 | 20.1 | 16.3 | 19.6 | 23.7 | 30.3 | 32.3 | 30.3 | 36.9 | 36.5 | 28.1 | 30.5 | 57.7 | 34.2 | 13.0 | |
| Current smoker | 22.3 | 21.7 | 21.6 | 29.9 | 20.6 | 29.1 |  | 18.7 | 20.1 | 27.6 | 20.0 | 31.1 | 25.3 | 28.0 | 36.2 | 21.4 | 26.9 | 18.5 | 10.2 | 3.9 | 21.1 | 21.7 | |
| **Living in urban/rural area, %** |  |  |  |  |  |  | 0.34 |  |  |  |  |  |  |  |  |  |  |  |  |  |  |  | |
| **N** | 5212 | 1967 | 2282 | 278 | 487 | 198 |  | 621 | 806 | 102 | 237 | 123 | 944 | 1021 | 150 | 209 | 52 | 402 | 455 | 26 | 41 | 23 | |
| Urban | 86.0 | 87.0 | 85.7 | 85.3 | 85.2 | 82.3 |  | 89.2 | 85.9 | 85.3 | 84.4 | 82.9 | 85.4 | 84.5 | 85.3 | 85.7 | 80.8 | 87.6 | 87.9 | 84.6 | 87.8 | 82.6 | |
| Rural | 14.0 | 13.0 | 14.3 | 14.8 | 14.8 | 17.7 |  | 10.8 | 14.1 | 14.7 | 15.6 | 17.1 | 14.6 | 15.5 | 14.7 | 14.4 | 19.2 | 12.4 | 12.1 | 15.4 | 12.2 | 17.4 | |
| **Feeling rooted in residential area, %** |  |  |  |  |  |  | 0.10 |  |  |  |  |  |  |  |  |  |  |  |  |  |  |  | |
| **N** | 5426 | 2039 | 2377 | 295 | 504 | 211 |  | 702 | 900 | 122 | 257 | 135 | 936 | 1025 | 146 | 208 | 53 | 401 | 452 | 27 | 39 | 23 | |
| Highly | 39.0 | 39.8 | 39.8 | 36.3 | 35.7 | 34.1 |  | 25.6 | 26.1 | 29.5 | 29.2 | 23.7 | 45.6 | 46.4 | 37.0 | 39.9 | 49.1 | 51.1 | 51.8 | 63.0 | 56.4 | 60.9 | |
| To some extent | 37.2 | 36.3 | 38.0 | 35.3 | 38.9 | 35.1 |  | 36.2 | 41.0 | 34.4 | 37.4 | 39.3 | 37.4 | 35.5 | 37.0 | 40.9 | 30.2 | 34.2 | 37.4 | 29.6 | 38.5 | 21.7 | |
| Not especially | 18.8 | 19.1 | 17.7 | 22.4 | 18.7 | 24.6 |  | 29.5 | 25.9 | 28.7 | 23.7 | 28.2 | 14.1 | 14.5 | 20.6 | 14.9 | 18.9 | 12.7 | 8.4 | 3.7 | 5.1 | 17.4 | |
| Not at all | 5.0 | 4.7 | 4.6 | 6.1 | 6.8 | 6.2 |  | 8.7 | 7.0 | 7.4 | 9.7 | 8.9 | 2.9 | 3.5 | 5.5 | 4.3 | 1.9 | 2.0 | 2.4 | 3.7 | 0.0 | 0.0 | |
| **Perceived Sensory Dimension score (PSD3-score, 3 items),** | 4949 | 1881 | 2154 | 272 | 455 | 187 |  | 598 | 763 | 99 | 223 | 117 | 904 | 964 | 147 | 195 | 48 | 379 | 427 | 26 | 37 | 22 | |
| **standardized, N** |  |  |  |  |  |  |  |  |  |  |  |  |  |  |  |  |  |  |  |  |  |  |  |
| **Perceived Sensory Dimension score (PSD3-score, 3 items),** | 0 | -0.03 | 0.01 | 0.03 | 0.02 | 0.02 | 0.66 | -0.19 | -0.10 | 0.05 | -0.03 | -0.06 | 0.07 | 0.11 | -0.02 | 0.09 | 0.25 | -0.02 | -0.001 | 0.20 | 0.03 | -0.04 | |
| **standardized, Mean** |  |  |  |  |  |  |  |  |  |  |  |  |  |  |  |  |  |  |  |  |  |  |  |
| SD | 1 | 1.00 | 1 | 0.96 | 0.99 | 1.06 |  | 1.00 | 1.02 | 1.01 | 1.05 | 1.09 | 1.00 | 1.00 | 0.98 | 0.92 | 1.02 | 1.00 | 0.93 | 0.69 | 0.94 | 0.95 | |
| Median | -0.16 | -0.20 | -0.13 | -0.12 | -0.15 | -0.20 |  | -0.39 | -0.29 | -0.01 | -0.22 | -0.28 | -0.14 | 0.03 | -0.16 | -0.08 | 0.13 | -0.17 | -0.05 | 0.18 | -0.05 | -0.21 | |

^1^p-value for comparison between classes using Chi-square test or linear regression as appropriate, excluding missing data.

SN: *Stable-normal*; SO: *Stable-overweight;* IO*: Increasing-obesity*; FOv: *Fluctuating-overweigh;,* FOb: *Fluctuating-obesity*
